# Prevalence and contributing factors of household food insecurity among women in Akinyele Local Government, Ibadan, Oyo State, Nigeria

**DOI:** 10.11604/pamj.2025.52.50.36958

**Published:** 2025-09-30

**Authors:** Olariike Oyindasola Kayode, Quadri Kunle Alabi

**Affiliations:** 1 Department of Human Nutrition and Dietetics, Osun State University, Osogbo, Nigeria,; 2 Department of Physiology, Faculty of Basic Medical Sciences, Adeleke University, Ede, Osun State, Nigeria

**Keywords:** Food insecurity, household, prevalence, women

## Abstract

**Introduction:**

food is paramount to a healthy life and should be adequate in terms of quantity and quality. A pressing social and public health issue is food insecurity, which has consequences on individuals, households, and the nation at large. The study aimed to determine the prevalence of food insecurity and its contributing factors among women in the Akinyele Local Government Area.

**Methods:**

this was a cross-sectional study conducted in the Akinyele Local Government Area, Ibadan. A pre-tested, structured interviewer-administered questionnaire was employed for collecting socioeconomic details and contributing factors to household food insecurity. Prevalence of food insecurity was assessed using the Household Food Insecurity Access Scale (HFIAS). Multistage sampling was used to select 399 households; out of 399 questionnaires distributed, 364 were retrieved, representing a 91.2% response rate. Chi-square was used to determine the prevalence of food insecurity and the association between food insecurity and sociodemographic characteristics of respondents.

**Results:**

the study revealed that a total of 76% of households were food-insecure, 11.6% of households were mildly food-insecure, 30.5% of households were moderately food-insecure, and 33.9% of households were severely food-insecure. There exists a significant association between socio-economic factors and food insecurity [X^2^=4.122; p-value< .036]. Family size was also found to be significantly associated with food insecurity [X^2^=2.889; p-value = 0.042]. Religion [X^2^=1.026; p-value = .117], Level of Education [X^2^=2.009; p-value = 0 .083] and Ethnicity [X^2^=1.361; p-value =0 .089] does not show significant association with food insecurity. Households with large family size were 5.5 times more likely (AOR = 5.5; 95% CI 1.06-17.15) to be food insecure compared to those with small family size.

**Conclusion:**

a high prevalence of food insecurity was reported among households in Akinyele Local Government Area, Ibadan. This requires prompt attention by policymakers to forestall the effects of food insecurity.

## Introduction

One of the basic requirements for the sustenance of life is food; it should be adequate in quantity and quality in order to live a healthy and productive life. Food security occurs when all people, at all times, have both physical and economic access to sufficient, safe, and nutritious food to meet their dietary needs and food preferences for an active and healthy life [1]. This can be examined at various levels, which include global, national, regional, household, and individual levels. Food security at the national or regional level does not translate to food security among communities, households, and individuals. Food security entails the availability of food, the accessibility (the physical and financial resources to access food), and the ability to utilize food [2].

Food insecurity occurs when there is irregular access to adequate, safe, and nutritious food for growth and development and to live an active and healthy life. This can be observed at different levels of severity, which are severe food insecurity, moderate, and mild food insecurity [3]. A severely food-insecure household has run out of food and gone a day or more without eating. Most times, the particular household has experienced hunger. At the household level, the presence of food insecurity suggests a high degree of vulnerability to a broad spectrum of consequences, including psychosocial dysfunction in children, socio-familial problems, and overall poor health status [4].

Food insecurity has both short and long-term consequences, which impact physical health, social, and economic development [5]. It often results in stress, cycles of fasting and bingeing, and the replacement of relatively higher cost, higher nutrition food with lower cost, energy-dense food, resulting in nutrient deficiencies, metabolic changes, weight loss, or, seemingly paradoxically, overweight and obesity [6].

Food insecurity is a global problem, though the prevalence and consequences are more severe in developing countries. Over 900 million people worldwide are chronically hungry, and of these, 800 million, representing about 18% of the world´s population, are in developing countries [7]. The Food and Agriculture Organization estimated that almost 200 million Africans were undernourished at the dawn of the millennium compared to 133 million 20 years earlier [7].

Lack of physical and economic access to safe and sufficient food by individuals and households has resulted in reactions and responses such as feelings of uncertainty or anxiety over food supply, reduction in portion sizes, and hunger [8]. The Household Food Insecurity Access Scale (HFIAS), which measures the access component of household food insecurity represented by the three domains of food insecurity, captures these experiences [9]. The Food Insecurity Access Scale (HFIAS), which measures whether a household is food insecure as well as the severity of the food insecurity, was used to determine the prevalence of food insecurity.

Furthermore, to the best of our knowledge, there is a dearth of information regarding food insecurity in the study area. Also, addressing food insecurity requires knowing prevalence of the problem and the contributing factors. The purpose of this study was to determine prevalence of food insecurity, socio-economic factors, and contributing factors to food insecurity among households in Akinyele Local Government Area, Ibadan, Nigeria.

## Methods

**Study area and design:** this study was conducted in the Akinyele Local Government Area. It occupies a land area of 464.892km^2^ with a population of 211,811 for a density of 516 persons per km^2^. There were 12 wards in Akinyele Local Government Area, these are: Ikereku, Olanla/Oboda/ Labode, Arulogun/Eniosa/Aroro, Olode/Amosun/ Onidundu, Ojo-Emo/Moniya, Akinyele/Isabiyi/ Irepodun, Iwokoto/Talonta/Idi-oro, Ojoo/Ajibode/ Laniba, Ijaye/ Ojedeji, Ajibade/ Alabata, Olorisa-Oko/Okegbemi/Mele, and Iroko. It was a cross-sectional study. The study was conducted between June and August 2021.

**Target population:** women aged 20-59 years who are living in Akinyele Local Government Area and were largely responsible for handling the food preparation and food distribution within the household were selected for the study.

**Ethical consideration:** the study was conducted according to the guidelines found in the Declaration of Helsinki. Ethical approval for the study was sought from the Ministry of Health, Oyo State, ethical review committee, with reference. no: AD/131479/4177. Respondents were duly informed about the purpose of the study, and written informed consent was obtained before the commencement of the study.

### Sampling technique and sample size determination

#### A multi-stage sampling technique was used

**Stage one:** random selection of three wards out of the twelve wards in Akinyele Local Government Area. Three (3) wards were selected by balloting; the wards selected were: Arulogun/Eniosa/Aroro, Olode/Amosun/Onidundu, and Ojo-Emo/Moniya.

**Stage two:** random selection of one community from the selected wards. The selected communities were Arulogun, Moniya, and Olode.

**Stage three:** 399 households were selected from the selected communities using systematic random sampling with a sampling interval of 5. At least 105 households were selected from each community, out of which 364 questionnaires were eventually used for the analysis.

### Sample size determination

Considering the prevalence of food insecurity in Edo State as 61.8%, a relative precision of 20%, a design effect of 2, and a non-response rate of 10%, the sample size was 399 [10].

### Operational definition

**Household:** a household consists of one or several persons who live in the same house and share meals [11].

**Food-secure:** a household was labelled ‘food-secure' when the members rarely, in the past four weeks, worried about not having enough food [9].

**Mildly food-insecure:** the members of the household worried about not having enough food sometimes or often, and/or were unable to eat preferred foods, and/or ate a more monotonous diet than desired, and/or ate some foods considered undesirable but only rarely [9].

**Moderately food-insecure:** the household members sacrificed quality more frequently by eating a monotonous diet or undesirable foods sometimes or often, and/or had started to cut back on quantity by reducing the size of meals or number of meals, rarely or sometimes [9].

**Severely food-insecure:** the individuals in the household had to cut back on meal size or number of meals often, and/or experienced any of the three most severe conditions (running out of food, going to bed hungry, or going a whole day and night without eating) [9].

**Instrument for data collection:** a pretested, structured questionnaire was used as an instrument for data collection. A 13-item household food insecurity access scale was used to determine the prevalence of food insecurity. The questionnaire was adapted from the Household Food Insecurity Access Scale developed by the Food and Agriculture Organization [12].

The questions contained in the Household Food Insecurity Access Scale (HFIAS) were asked with a recall period of four weeks (28 days). Respondents were asked an occurrence question, to state whether the condition in the question happened in the past month (yes or no response was given). Each of the questions was followed by frequency of occurrence to determine whether the condition happened rarely (once or twice), sometimes (3 to 10 times), often (11 to 20 times), or always (more than 20 times) in a month. The questionnaire for data collection was translated into the local language (Yoruba language).

**Data analysis:** the questionnaire contained 13 items on household food insecurity. A score was allocated for each question as follows: 3 if the statement never occurred, 2 if it occurred sometimes, and 1 if it occurred often. The respondents were classified into the categories of food insecurity based on their total score on the food insecurity scale. Households with a mean score of four were classified as food secure, households with mean score of three as mildly food insecure, households with mean score of two as moderately food insecure, and households with a mean score of one were classified as severely food insecure. Data were analyzed using Statistical Package for Social Sciences version 23. Chi-squared test was conducted to determine the association between socio-demographic characteristics and food insecurity, while multivariate logistic regression analysis was performed to determine the factors associated with food insecurity. A p-value of <0.05 was considered to be statistically significant.

## Results

Table 1 shows the socio-demographic characteristics of the respondents. Age results revealed that 9.9% were 20-29 years old, 47.8% were 30-39 years old, 33.0% were 40-49%, while 9.3% were 50 years and above. For religion, 66.5% were Christian, and 33.5% were Muslim. On the level of education, 48.4% had no formal education, 39.6% had primary school education, and 12.0% had secondary school education. On marital status, 28.6% were single, 66.5% were married, 0.5% were divorced, and 4.4% were widowed.

**Table 1:** socio-demographic characteristics of women in Akinyele Local Government, Ibadan

Items	Frequency	Percentage
**Age**	-	-
20-29	36	9.9
30-39	174	47.8
40-49	120	33.0
50 and above	34	9.3
**Religion**	-	-
Christianity	242	66.5
Islam	122	33.5
Traditional	0	0.0
**Level of Education**	**-**	**-**
No formal educational	176	48.4
Primary	144	39.6
Secondary	44	12.0
Tertiary	0	0.0
**Marital Status**	**-**	**-**
Single	104	28.6
Married	242	66.5
Divorced	2	0.5
Widowed	16	4.4
**Ethnici**ty	-	-
Yoruba	274	75.3
Hausa	2	0.5
Igbo	88	24.2
**Type of family**	-	-
Nuclear	320	87.9
Extended	44	12.1
**Occupation**	-	-
Teacher	44	12.1
Traders	130	35.7
Farmer	8	2.2
Others	182	50.0

**Figure 1:**
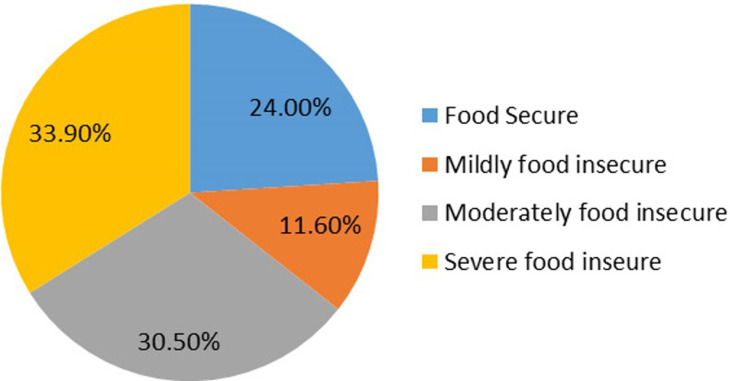
prevalence of food insecurity among women in Akinyele Local Government, Ibadan

**Prevalence of household food insecurity among the respondents:** as shown in Table 2 and Table 3, almost a quarter (24.2%) of the respondents eat once during the day, a majority (71.4%) eat twice, only 3.8% eat thrice in a day and 0.6% eat more than three times in a day. Most (70.9%) of the respondents worried that their household would not have enough food, while 29.1% of the respondents didn't worry about food. Among those who worry, 15.1% do that rarely, a third (33.4%) do that sometimes, 20.9% do that often, and almost a third (30.6%) have to worry about food always. Majority (69.8%) of the respondents reported that in the past four weeks, some household members had to eat a smaller portion of a meal than they felt was needed because there was not sufficient food, while 26.9% didn't. Among those who said yes, 12.2% said it rarely happens, 37.8% said it happens sometimes, 28.0% said it happens often, and 22.0% said it happens always. Majority (78.9%) of the respondents reported that some household members skipped a meal in the last four weeks because there was not enough money to buy food, 20.8% declined, and 10.2% were unsure. A larger percentage (76.1%) of the respondents said that there was no food to eat of any kind in their household in the past month because of a lack of money to get food, while 13.9% declined. Figure 1 shows that 24% were food secured, while 76.1% were food insecure (11.6% were mildly food insecure, 30.5% were moderately food insecure, and 33.9% were severely food insecure. Socio-economic factors contributing to food insecurity among the respondents as shown in Table 4, 48.3% of the respondents reported that family earnings are no longer sufficient to buy enough food, while 41.2% of the respondents reported that family size is too large and food is being rationed.

**Table 2:** prevalence of household food insecurity status among women in Akinyele Local Government, Ibadan

Items	Frequency	Percentage
**How many times do you eat during the day?**	-	-
Ones	88	24.2
Twice	260	71.4
Three times	14	3.8
More than three times	2	6
**How many times do you or any member of your family go to market to buy foodstuff for your household?**		
Daily	122	33.5
One’s a week	82	22.5
Twice a week	38	10.4
Three or more times a week	81	22.3
Monthly	41	11.3
**In the past 4 weeks, did you worry that your household would not have enough food?**	**-**	**-**
Yes	258	70.9
No	106	29.1
If yes, how often did this happen?	-	-
Rarely (1-2 times)	39	15.1
Sometimes	86	33.4
Often	54	20.9
Always	79	30.6
**In the past 4 weeks, were you or any of your household members not able to eat the kind of food you preferred because of a lack of resources?**	-	-
Yes	266	73.1
No	98	26.9
If yes, how often did this happen?	-	-
Rarely	38	14.3
Sometimes	29	10.9
Often	71	26.7
Always	128	48.1
**Did you or any household member have to eat a limited variety of foods due to lack of resources?**	-	-
Yes	254	69.8
No	110	30.2
If yes, how often did this happen?	-	-
Rarely	31	12.2
Sometimes	96	37.8
Often	71	28.0
Always	56	22.0
**In the past 4 weeks, did you or any household member have to eat a smaller meal than you felt you needed because there was not enough food?**	-	-
Yes	254	69.8
No	110	30.2
If yes, how often did this happen?	-	**-**
Rarely	31	12.2
Sometimes	96	37.8
Often	71	28.0
Always	56	22.0

**Table 3:** prevalence of household food insecurity status among women in Akinyele Local Government, Ibadan

Items	Frequency	Percentage
**In the last 4 weeks, have there been any time you went to bed without eating?**	**-**	**-**
Yes	114	31.3
No	194	53.3
Not sure	56	15.4
**If yes, why?**	**-**	**-**
Food was not available	88	77.2
Tired to cook	19	16.7
Slept off before food was ready	7	6.1
**In the last 4 weeks, have there been any day you left home in the morning without eating?**	**-**	**-**
Yes	180	49.5
No	110	30.2
Not sure	74	20.3
If yes why?	**-**	**-**
Food was not available	93	51.7
There was no time	42	23.3
Food was not ready	45	25.0
**In the last 4 weeks, do you or any other household members ever skip a meal because there was no enough money to buy food?**	**-**	**-**
Yes	287	78.9
No	58	15.9
Not sure	19	5.2
**Sometimes people lose weight because of not having enough food to eat, in the last 30 days do you lose weight due to not enough food?**	**-**	**-**
Yes	251	69.0
No	76	20.8
Not sure	37	10.2
**In the past 4 weeks, did you or any other household member have to eat fewer meals in a day because there was not enough food?**	**-**	**-**
Yes	318	87.4
No	46	12.6
If yes, how often?	**-**	**-**
Rarely	23	7.2
Sometimes	79	24.9
Often	23	7.2
Always	193	60.7
**In the past 4 weeks was there ever no food to eat of any kind in your household because of lack of resources to get food?**	**-**	**-**
Yes	277	76.1
No	87	13.9
If yes, how often did this happen?	**-**	**-**
Rarely	19	6.9
Sometimes	59	21.3
Often	31	11.2
Always	168	60.6

**Table 4:** socio-economic factors contributing to food insecurity among women in Akinyele Local Government, Ibadan

S/N	Items	AA	SA	NA
1.	Our family's earnings are no longer sufficient to buy enough food	176 (48.3)	132 (36.3)	56 (15.4)
2.	Our family size is too large, so we ration our food	150 (41.2)	112 (30.8)	102 (28.0)
3.	Certain food items are taboo to use, so regardless of availability, we don’t eat those foods	36 (9.9)	106 (29.1)	222 (61.0)
4.	I can’t do without being online or having recharge cards per day, even at the expense of food	152 (41.8)	146 (40.1)	66 (18.1)
5.	My husband must visit the bar at least three times a week	88 (24.2)	176 (48.3)	100 (27.5)
6.	I love attending occasions/ buying “Aso-Ebi”	202 (55.5)	94 (25.8)	68 (18.7)
7.	Who to cook food used to be a prominent problem in my family, so individuals often settle for fast food (such as garri) often	24 (6.5)	60 (16.5)	280 (77.0)

AA: always applicable, SA: sometimes applicable, N: not applicable

**Association between socio-demographic characteristics and prevalence of food insecurity among the respondents:** Table 5 shows that, Age [X=3.409; p-value < .012]; Marital Status [X=2.676; p-value < .018]; Type of family [X=2.889; p-value < .042] and Occupation [X=2.791; p-value < .034] have significant relationship with food insecurity. However, Religion [X=1.026; p-value > .117], Level of education [X=2.009; p-value < .083], and Ethnicity [X=1.361; p-value < .089] do not have a significant relationship with food insecurity status.

**Table 5:** association between socio-demographic characteristics and prevalence of food insecurity

Items	Frequency	Food Secure (87)	Food Insecure (277)	X2	P-value
**Age**	-	-	-	-	-
20-29 years	36	16	20	3.409	012
30-39 years	174	22	152	-	-
40-49 years	120	38	82	-	-
50 years & above	34	11	23	-	-
**Religion**	-	-	-	-	-
Christianity	242	58	184	1.026	117
Islam	122	29	73	-	-
**Level of education**	**-**	**-**	**-**	**-**	-
No formal education	176	43	133	2.009	083
Primary	144	35	109	-	-
Secondary	44	9	35	-	-
**Marital status**	**-**	**-**	**-**	**-**	**-**
Single	104	36	68	2.676	.018
Married	242	38	204	-	-
Divorces	2	1	1	-	-
Widowed	16	12	4	-	-
**Ethnic**ity	-	-	-	-	-
Yoruba	274	67	207	1.361	.089
Hausa	2	1	1	-	-
Igbo	88	19	69	-	-
**Type of family**	-	-	-	-	-
Nuclear	320	55	265	2.889	.042
Extended	44	32	12	-	-
**Occupation**	-	-	-	-	-
Teacher	44	12	32	2.791	.034
Traders	130	38	92	-	-
Farmer	8	3	5	-	-
Others	182	34	148	-	-

**Factors associated with household food insecurity among the respondents:** as shown in Table 6, family earnings, family size, and buying aso ebi were shown to be the factors associated with food insecurity among the respondents.

**Table 6:** logistic regression analysis of factors associated with household food insecurity among women in Akinyele Local Government, Ibadan

	Crude OR (95% CI)	Adjusted OR (95%CI)	P- value
Marital status	-	-	-
Single	Ref	-	-
Married	0.48(0.09-2.64)	-	0.58
Divorced	0.49(0.69-1.45)	-	-
Widowed	0.99(0.64-1.49)	-	-
Family earning	-	-	-
Sufficient	0.97(0.13-6.94)	0.39(0.05-3.03)	-
Insufficient	0.48(0.25-0.95)	0.29(0.14-0.63)	**0.01**
Maternal occupation	-	-	-
Teacher	0.84(0.44-1.49)	-	-
Trader	0.42(0.27-0.73)	-	0.47
Farmer	0.91(0.13-2.14)	-	-
Others	0.49(0.29-0.58)	-	-
Family size	-	-	-
Small	0.44(0.27-0.73)	0.39(0.22-0.67)	-
Large	4.38(0.95-11.16)	5.5(1.06-17.15)	**0.021**
Attending occasion/buying Aso ebi	-	-	-
Yes	0.51(0.29-0.87)	0.43(0.24-0.80)	-
no	0.82(0.54-1.24)	1.34(0.84-2.13)	**0.03**

## Discussion

The majority (76%) of the households were food insecure, with 33.9% being severely food insecure, which shows that most respondents cannot afford three meals per day due to lack of funds and unavailability of food at home, and have to cut back on meal size. The plausible reason for this is that there is an economic recession in Nigeria, which has adversely affected the means of livelihood of many households. The level of food insecurity observed in the current study is in contrast with previous study carried out in Osun State, it was reported that the prevalence of food insecurity was 54% [13] but is in agreement with findings from a study carry out in Imo State where the prevalence of food insecurity was reported to be 79% [14]. The high prevalence of food insecurity observed among the studied households is cause for concern, and encourages immediate action to be taken to forestall the adverse effects this might have on the household members.

The study revealed that level of education was not significantly associated with food insecurity [X^2^=2.009; p-value = 0.083], this was in disagreement with a study carried out in Ethiopia, which reported that the head of households with a low level of education had a higher risk of being food insecure [15]. However, being educated (going to school) does not translate to financial education, which is not taught in school.

This finding revealed that having a large family size is significantly associated with food insecurity [X^2^=2.889; p-value = 0.042]. This study is supported by the studies done in Nigeria and other African Countries [16-18], which reported food insecurity in a family of large size. This may be because larger families have a higher chance of possessing a lower per capita income, which in turn affects the portion size of food distributed to members of the household. This finding calls for health education on the importance of family planning for reduction in family size.

Findings also revealed that socio-economic factors militating against food security include insufficient family earnings, large family size, and preference for other things over food. Almost half (48.3%) of the households reported that the family earnings are insufficient and cannot afford sufficient food for the members of the household. More than half (55.5%) of the respondents reported buying expensive clothes for a party at the expense of food.

The study revealed a significant association between socio-economic status and food insecurity. Poor households (66.7%) had a higher risk of food insecurity compared to rich households (1.4%), which corroborates with other findings that low income is the major and most consistent predictor of food insecurity, especially in developing countries [19]. Also, high prices of food have made the situation worse and resulted in restrictions on the portion and quality of meals consumed, reduced dietary variety, and consumption of inexpensive processed food. Logistic regression analysis shows that family size, family earnings, and attending occasions/buying asoebi were factors contributing to household food insecurity. Households with large family size were 5.5 times more likely (AOR=5.5; 95% CI=1.06-17.15) to be food insecure compared to those with small family size.

The limitation of this study includes recall bias, as the HFIAS tool sought to ascertain past experiences of food insecurity. Also, the quality of food consumed and gender discrimination in food allocation were not considered. These aspects would need to be explored in future studies.

## Conclusion

The study revealed a high prevalence of food insecurity among households in the Akinyele Local Government Area, Ibadan. Identified factors militating against food security include insufficient family earnings, large family size, and preference for other things over food. The governments need to make strategies to help quick economic recovery to forestall the effects of a financial crisis and ensure women´s empowerment. Public health experts, through health education, need to encourage the masses to embrace the use of family planning methods for population control. Household heads should ensure that the purchase of food is prioritized over non-food items.

### What is known about this topic

- Food security should be examined at various levels: global, national, regional, household, and individual levels;
- Food security at the national or regional level does not translate to food security at the household level;
- Food insecurity can be either mild, moderate, or severe.

### What this study adds

- Poor households have a higher risk of food insecurity compared to rich households;
- Factors militating against food security include insufficient family earnings, large family size, and preference for clothes over food;
- The study revealed that the level of education was not significantly associated with food insecurity.

## References

[1] Food and Agriculture Organisation (2003). Food security: concepts and measurement. Food and Agriculture Organisation. Trade reforms and food security: conceptualizing the links.

[2] Kleve S, Gallegos D, Ashby S, Palermo C, McKechnie R (2018). Preliminary validation and piloting of a comprehensive measure of household food security in Australia. Public Health Nutr.

[3] Bawadi HA, Tayyem RF, Dwairy AN, Al-Akour N (2012). Prevalence of food insecurity among women in northern Jordan. J Health Popul Nutr.

[4] Chinnakali P, Upadhyay RP, Shokeen D, Singh K, Kaur M, Singh AK (2014). Prevalence of household-level food insecurity and its determinants in an urban resettlement colony in north India. J Health Popul Nutr.

[5] Booth S, Smith A (2001). Food security and poverty in Australia-challenges for dietitians. Australian Journal of Nutrition and Dietetics.

[6] Ramsey R, Giskes K, Turrell G, Gallegos D (2012). Food insecurity among adults residing in disadvantaged urban areas: potential health and dietary consequences. Public Health Nutr.

[7] FAO (2008). The state of food security in the world.

[8] Food and Nutrition Technical Assistance (FANTA) Project (2004). Measuring Household Food Insecurity workshop. Workshop Report.

[9] Coates J, Swindale A, Bilinsky P (2007). Household Food Insecurity Access Scale (HFIAS) for measurement of food access: indicator guide: version 3. Washington D.C: Food and Nutrition Technical Assistance (FANTA) Project, Academy for Educational Development.

[10] Omuemu VO, Otasowie EM, Onyiriuka U (2012). Prevalence of food insecurity in Egor local government area of Edo State, Nigeria. Ann Afr Med.

[11] William A (2003). Haviland. Anthropology Wasword/Thomson Learning.

[12] Food and Agriculture Organization of the United Nations 2013 Statistics Division (ESS). Technical Paper Rome, FAO.

[13] Fawole WO, Ozkan B, Ayanrinde FA (2016). Measuring food security status among households in Osun State, Nigeria. British Food Journal.

[14] Nnakwe N, Onyemaobi G (2013). Prevalence of Food Insecurity and Inadequate Dietary Pattern Among Households with and without Children in Imo State Nigeria. International Journal of Sociology and Anthropology.

[15] Ayele AW, Kassa M, Fentahun Y, Edmealem H (2020). Prevalence and associated factors for rural households food insecurity in selected districts of east Gojjam zone, northern Ethiopia: cross-sectional study. BMC Public Health.

[16] Olayemi AO (2012). Effects of family size on household food security in Osun State, Nigeria. Asian journal of agriculture and rural development.

[17] Sekhampu TJ Determinants of the food security status of households receiving government grants in Kwakwatsi. South Africa.

[18] Shone M, Demissie T, Yohannis M (2017). Household food insecurity and associated factors in West Abaya district, southern Ethiopia. Agric Food Secur.

[19] Seivwright AN, Callis Z, Flatau P (2020). Food Insecurity and Socioeconomic Disadvantage in Australia. Int J Environ Res Public Health.

